# Spectral CT in peritoneal carcinomatosis from ovarian cancer: a tool for differential diagnosis of small nodules?

**DOI:** 10.1186/s41747-022-00302-z

**Published:** 2022-10-17

**Authors:** Giada Zorzetto, Andrea Coppola, Valeria Molinelli, Maria Gloria Angeretti, Jvan Casarin, Federico Fontana, Filippo Piacentino, Giulio Carcano, Fabio Ghezzi, Massimo Venturini

**Affiliations:** 1 Diagnostic and Interventional Radiology Department, Circolo Hospital — ASST dei Sette Laghi, University of Insubria, Varese, Italy; 2 Obstetrics and Gynecology Department, Filippo Del Ponte Hospital — ASST dei Sette Laghi, University of Insubria, Varese, Italy; 3 General, Emergency and Transplants Surgery Department, Circolo Hospital — ASST dei Sette Laghi, University of Insubria, Varese, Italy

**Keywords:** Cytoreduction surgical procedures, Diagnosis (differential), Ovarian neoplasm, Peritoneal neoplasms, Tomography (x-ray computed)

## Abstract

The detection of peritoneal carcinomatosis in patients with ovarian cancer is crucial to establish the correct therapeutic planning (debulking surgery *versus* neoadjuvant chemotherapy).

Often, however, the nodules of peritoneal carcinomatosis are very small in size or have a reticular appearance that can mimic the fat stranding that is typical of acute inflammation conditions. Our hypothesis is that the use of dual-layer spectral computed tomography with its applications, such as virtual monoenergetic imaging and Z-effective imaging, might improve the detection and the characterisation of peritoneal nodules, increasing sensitivity and diagnostic accuracy, as recently described for other oncological diseases.

## Key points

- Peritoneal carcinomatosis detection is crucial to define a correct therapeutic planning.
- Dual-layer detector spectral computed tomography may improve peritoneal carcinomatosis detection in ovarian cancer.
- Virtual monoenergetic and Z-effective reconstructions may allow to diagnose small peritoneal nodules.

Peritoneal carcinomatosis greatly influences prognosis and outcome of patients with advanced abdominopelvic malignancy including ovarian, gastric, colorectal, pancreatic, and adrenocortical carcinomas [1, 2]. In particular, the detection of peritoneal carcinomatosis plays an important role in ovarian cancer, which represents the second most common gynecological malignancy and the first cause of death due to gynecological causes in women. Ovarian cancer presents in an advanced stage (stages 3C or 4 according to the International Federation of Gynecology and Obstetrics, FIGO) in 70% of patients at the time of diagnosis, with a 5-year survival rate of only 25−37% [3, 4].

In patients with ovarian cancer, a correct identification of the peritoneal spread is therefore essential to establish the correct therapeutic choice between upfront cytoreductive surgery (debulking) followed by adjuvant chemotherapy or neoadjuvant chemotherapy and subsequent interval debulking surgery [5, 6]. Intraoperative evaluation of the peritoneal carcinomatosis index is still nowadays the standard of care for peritoneal carcinomatosis detection and quantification of extension, given the poor sensitivity of standard imaging methods [5].

Different imaging techniques such as ultrasound [7], computed tomography (CT) [8, 9], positron emission tomography/CT [10], and magnetic resonance imaging (MRI) [11] have been employed to detect peritoneal carcinomatosis from ovarian and gastrointestinal cancer [12]. In a recent published meta-analysis [13], the pooled sensitivity, specificity, and diagnostic odds ratio were reported to be as follows: 68%, 88%, and 15.9 for CT; 80%, 90%, and 36.5 for positron emission tomography/CT; and 92%, 85%, and 63.3 for diffusion-weighted MRI, respectively.

CT remains the modality of choice for the pretreatment evaluation of patients with ovarian cancer with an overall accuracy from 70 to 90%, especially for the evaluation of those abdominal areas that are difficult to reach and to identify with explorative laparotomy or laparoscopy (*i.e.,* diaphragm, stomach, splenic and hepatic hila, mesenteric root, and suprarenal para-aortic lymph nodes) which might represent a criterion for defining a condition of nonresectable disease [13]. The combination of preoperative CT and intraoperative laparoscopy with the calculation of the peritoneal carcinomatosis index [14] can facilitate an accurate diagnosis, although it is still difficult to safely refer patients to debulking surgery or neoadjuvant chemotherapy.

The major limitation of CT is represented by the difficult detection of very small nodules of peritoneal carcinomatosis with a diameter of less than 5 mm located on the peritoneum, omentum, or intestinal serosa and by the complex differential diagnosis with other peritoneal diseases, such as peritoneal fibrosis, benign lymphadenomegaly, or “fat stranding” in inflammatory pathologies [15]. The differentiation between omental fat stranding and clustered subcentimeter nodules may be particularly complex [16].

To overcome this problem, the use of MRI has been recently proposed for the evaluation of peritoneal carcinomatosis [17]. This technique is comparable to CT but with a higher sensitivity, thanks also to the use of diffusion-weighted images [18–20]. MRI is characterised by a high soft-tissue contrast with consequent elevated capability to differentiate tumours from the normal tissue, although it is well known to have a lower spatial resolution than CT [19]. Moreover, MRI does not allow to correctly evaluate the diaphragm and the pericardiophrenic regions as well as the small implants on the wall of the small intestine, due to the image quality degradation from cardiac pulsatility and magnetic susceptibility artifacts, which invalidate the evaluation of the air-soft tissue interface areas. Furthermore, another limitation of MRI is represented by the poor precision of the anatomical information given by the diffusion-weighted sequences. To these, disadvantages must also be added to all the problems in case of patients with contraindications to MRI (such as unsafe intracardiac devices, intraocular splinters, cochlear implants, tattoos in the body region to investigate, claustrophobia), which do not make it accessible to all patients [21, 22].

In this context, the limits of MRI and conventional CT might be overcome by spectral CT.

Conventional CT using a single x-ray energy has well-known limitations in terms of contrast resolution among soft tissues. Dual-energy (and multi-energy) spectral CT can better discriminate different tissues by exploiting their energy-dependent attenuation properties. There are different “spectral” technologies that are becoming more and more available for clinical practice, such as fast kilovoltage-switching CT, split filter CT, dual-source CT, and dual-layer CT. In a recent study, Graffier et al. [23] evaluated the performance of these cutting-edge technologies. Through a task-based image quality assessment of noise characteristics, spatial resolution, and lesion detection using virtual monoenergetic imaging (VMI) at low-energy levels, they found that dual-layer CT offered better detection of small lesions even at values of 40−50 keV, lower than kilovoltage-switching or dual-source CT (60−80 keV).

Dual-layer detector CT can simultaneously acquire two polychromatic datasets corresponding to two different mean-energy photons. The top layer (yttrium-based garnet scintillator) selectively absorbs low-energy photons, while the bottom layer (gadolinium oxysulphide-based scintillator) selectively absorbs high-energy photons. Attached to each layer, a photodiode converts light into an analogue electrical signal, and an application-specific integrated circuit converts it into a digital signal [24]. The images obtained can be converted into different types of spectral imaging: iodine-density imaging, virtual non-contrast imaging, virtual monoenergetic imaging, effective atomic number (Z-effective imaging), and uric acid pair imaging. These images allow an enhanced visualisation of vascular contrast as well as artifact reduction, material decomposition, and, lastly, radiation dose reduction [24].

VMI is derived and reconstructed from dual-energy CT data at a chosen keV level, without additional radiation exposure or further contrast agent administration. Through VMI, it is possible to obtain low-energy (40–70 keV) reconstructions that enhance the visualisation of intravascular contrast, thanks to the improvement of iodine attenuation at lower keV levels. This can enhance the visualisation and provide a better differentiation of small vascularised lesions with a lower dose of contrast agent, providing a better conspicuity of oncological abnormalities [25].

Another reconstruction image whose clinical and diagnostic utility could be clinically relevant is the Z-effective imaging, a colour-coded representation of the effective atomic number (Z) of different tissues. The greater the atomic number of the main constituent element of the examined structure, the closer the colour on the image will be to blue (*e.g.,* iodine, bone). As the atomic number decreases, it changes to green-yellow and finally to red (*e.g.,* muscle, soft tissues). Through these reconstructions, dual-layer spectral CT has in fact some more ability to distinguish the presence of calcium, adipose tissue, or water components within the lesion.

These techniques can be applied in multiple ways to add clinical value in the diagnosis and management of peritoneal carcinomatosis in patients with ovarian cancer. Pang et al. [26] have already published a study concerning the evaluation of spectral CT effectiveness in the differential diagnosis between malignant and benign ovarian tumours, referring to larger masses [26].

A spectral CT with dual detector (IQon, Philips Healthcare, Best, the Netherlands) was installed in our centre in March 2021. In our hypothesis, we intend to use all the features of the dual-layer spectral CT, especially VMI and the Z-effective imaging, to improve the detection of small nodules, with a diameter of less than 5 mm, especially in those surgically difficult-to-reach sites (diaphragm, mesentery root, etc.), without the need to submit the patients to a long and expensive examination such as MRI. Moreover, spectral CT might also be helpful in the characterisation of peritoneal nodules, as already proposed by Thivolet et al. [12] in a rat model of colorectal cancer, and in the differential diagnosis between carcinomatosis, fibrosis, physiologic lymph nodes, or pathological lymphadenopathies [27].

**Fig. 1:**
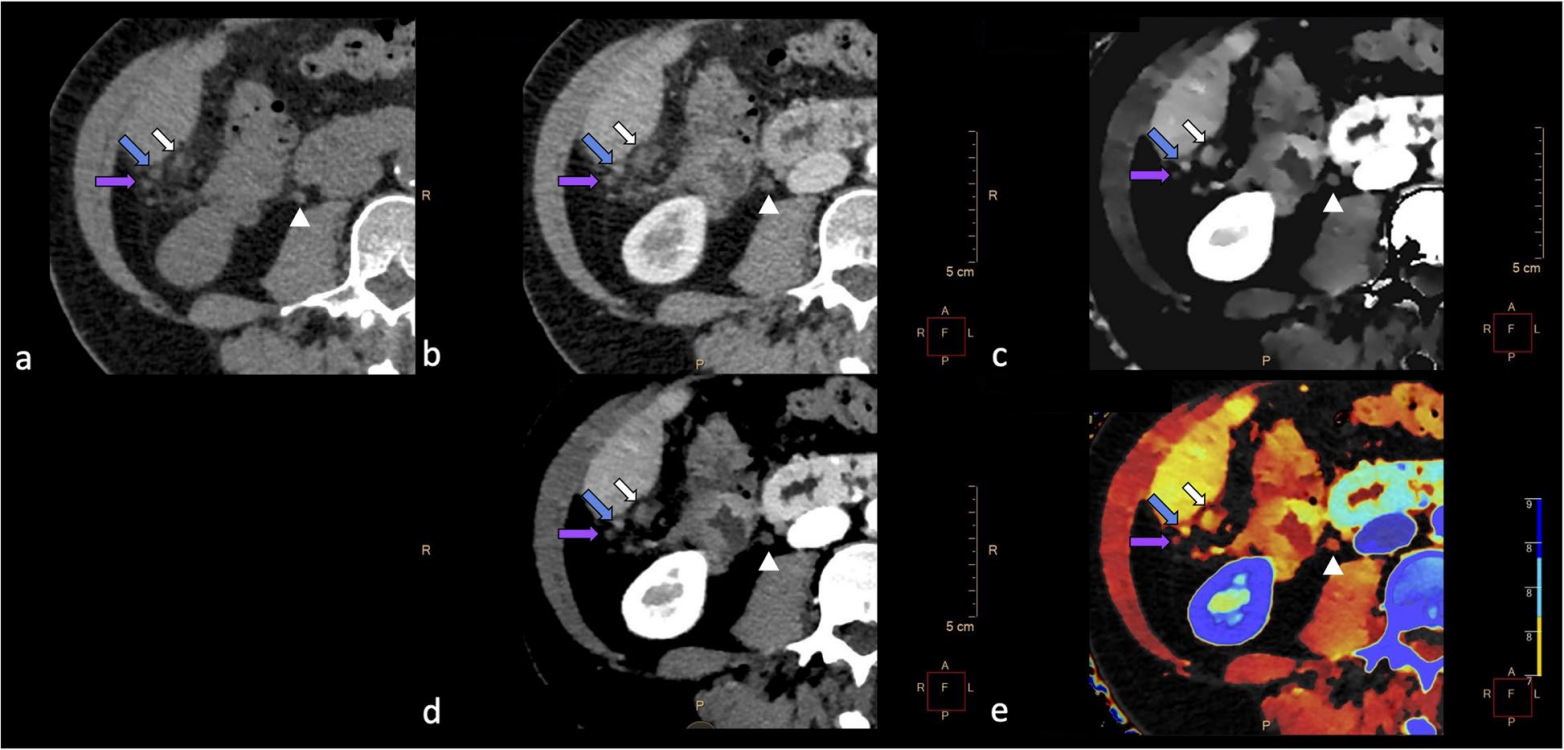
**a**–**e** Spectral computed tomography of a 64-year-old woman with peritoneal carcinomatosis from ovarian cancer. In the native CT scan, obtained before the intravenous administration of contrast agent (**a**) and in the portal-venous phase (**b**), two small and almost apparently identical subhepatic nodules (purple and blue arrows) and a large peritoneal carcinomatosis lesion (white arrow) can be seen. Please also note a small paracaval lymph node (arrowhead), benign in appearance. Iodine density map (**c**), 40 keV monoenergetic image (**d**), and Z-effective image (**e**), obtained at the same level of image **b**; the two small subhepatic nodules present different appearances. While the blue arrow nodule is similar to the large peritoneal carcinomatosis lesion (white arrow), the purple arrow nodule is similar to the small benign para-caval lymph node (arrowhead). This could suggest a different nature of these nodes: the blue arrow one is more likely to be a peritoneal carcinomatosis node, and the purple arrow node is instead more likely to be a small peritoneal lymph node or a fibrotic nodule

In a patient affected by ovarian cancer with ascites and suspected peritoneal carcinomatosis, two subhepatic nodules with the same morphological and densitometric characteristics at conventional CT (medium density of 10 HU and 13 HU at unenhanced images, of 40 HU and 75 HU at contrast-enhanced images) showed a different iodine concentration in the iodine density maps (1.7 *versus* 3.1 mg/L) and a different density at VMI (175 HU *versus* 257 HU), enhancing the visualisation of pre-existing density difference [28, 29]. Moreover, in the postprocessing reconstruction, VMI showed a different contrast enhancement between the two nodules, while the Z-effective imaging differentiated the two nodules in the colour-coded map as yellow and red, respectively. This could mean that the yellow nodule may contain structures with a higher atomic number than the red nodule, due to different tissue composition. Interestingly, in the same slice, a large peritoneal carcinomatous mass was evident, which presented spectral characteristic similar to the yellow nodule, while a paracaval lymph node, benign in appearance, presented spectral characteristic similar to the red nodule (Fig. 1). Our interpretation is that the hypervascular nodule with high atomic number is to be referred to peritoneal carcinomatosis, while the hypovascular nodule with low atomic number is to be considered as benign finding (lymph node or fibrosis).

Recent studies have emphasised the use of VMI reconstructions at 40 keV [30] as well as the association of iodine overlays with conventional images [31] to improve the diagnostic confidence for the diagnosis of peritoneal carcinomatosis. According to our hypothesis, the reconstruction with Z-effective imaging, providing a different colour-coded map, weights both the attenuation and atomic number of certain tissue, thereby facilitating the identification of predominant materials within the nodule.

However, sensitivity and diagnostic accuracy of dual-layer spectral CT compared to conventional CT methods or MRI must be investigated on a cohort of patients with ovarian cancer. Moreover, the results obtained should be validated by the surgical and histopathological data.

## Data Availability

Not applicable.

## Abbreviations

CT: Computed tomography
MRI: Magnetic resonance imaging
VMI: Virtual monoenergetic imaging
